# Characterization of fin whale song off the Western Antarctic Peninsula

**DOI:** 10.1371/journal.pone.0264214

**Published:** 2022-03-10

**Authors:** Megan Wood, Ana Širović

**Affiliations:** 1 Department of Marine Biology, Texas A&M University at Galveston, Galveston, TX, United States of America; 2 Biology Department, Norwegian University of Science and Technology, Trondheim, Norway; Institute of Deep-sea Science and Engineering, Chinese Academy of Sciences, CHINA

## Abstract

Song is produced by a variety of terrestrial and marine animals and is particularly common among baleen whales. Fin whale (*Balaenoptera physalus*) song is comprised of relatively simple 20 Hz pulses produced at regular intervals. The timing of these intervals, in addition to the presence and frequency of overtones, appears to be unique to each population. The purpose of this study was to characterize Western Antarctic Peninsula fin whale song and describe temporal pattern variations in song type and occurrence. Recordings were collected in the area from 2001–2004 and again 2014–2016. One song type was identified with a primary inter-pulse interval (IPI) of approximately 14 s and secondary IPI of 12.5 s. This song occurred in three pattern variants: singlet, doublet, and long triplet. The interval between pulses increased by 1.5 s between recording periods while the frequency of the overtones decreased from 89 Hz to 86 Hz. Song was never recorded in August and while it was recorded at other times in some years, it was consistently present in recordings from April through June across all years. While multiple pattern variants were present each year, singlets were generally the most prevalent variant. Doublets and triplets occurred from February through June, with highest levels of variants in February. In later years the triplet variant presence increased and in 2016 it comprised 53% of recorded song bouts. Further research is needed to understand the reasons why song changes over time and to examine the feasibility of using song to delineate and identify populations.

## Introduction

Auditory signals are used throughout the animal kingdom to communicate with conspecifics. Sound allows individuals to convey information across great distances and in environments with poor visibility [1]. When these sounds are stereotyped in nature and produced in patterned sequences they are called song; songs have been most well documented in birds [2, 3], but are also produced by frogs [4], mice [5], bats [6], fish [7], and cetaceans [8–10]. While the exact function of these songs is unclear and may vary across taxa, the generally observed concentration of song during mating periods argues a function in sexual selection and reproduction [11].

Songs can be innate or learned from conspecifics [12, 13]. Social learning has been well-described in birds, bats, primates and cetaceans [3, 11–15]. Learned songs are plastic over time as individuals change their vocalizations based on interactions with other individuals [3, 12, 13]. In different taxa this has resulted in regional dialects, or songs which are unique to a specific area or genetic population [8, 10, 16–22].

Several baleen whale species produce species-specific low-frequency songs which vary in their complexity. Humpback whale (*Megaptera novaeangliae*) songs are arguably the most complex [10, 23], blue whales (*Balaenoptera musculus*) produce relatively simpler songs combining pulsed and tonal units [8], while fin whale (*B*. *physalus*) songs appear the least complex as they consist of simple pulses [24]. The complexity of fin whale song, however, lies in the metronomic rates at which those pulses are produced, as well as the patterning of pulses of different bandwidth [21]. In these three species only males have been observed to engage in singing behavior [25–27]. Additionally, each species exhibits geographic variation in song which has been used to delineate populations [8, 17–21, 23, 28].

Baleen whale song has been observed to undergo change over time. In some species, such as the humpback whale, song changes over the course of a season and these accumulated innovations can render the song entirely unrecognizable from the original within years [10, 15, 18, 23, 25, 29]. In species with more stable songs, however, these changes are often more limited. Across ocean basins, a downward shift in song frequency has been observed in populations of blue whales [30–33], bowhead whales (*Balaena mysticetus*) [34], and fin whales [31, 35]. In fin whales, inter-pulse interval (IPI) values have been observed to lengthen over time [16, 35, 36] and evidence of sudden changes in song IPI have been found as well [16, 37]. In order to use song as a population indicator, it is important to understand the song changes to understand when a new song is indicative of a change in song preference or the presence of a new population.

Fin whale song is comprised of a series of short 1 s notes or pulses that are centered around 20 Hz and are referred to as 20 Hz pulses or 20 Hz notes [24, 38]. These pulses are sometimes accompanied by higher frequency components that are not harmonically related to the fundamental of the signal, i.e. overtones. While the frequency of these overtones differs among populations, it appears to be relatively consistent over time [20, 28, 39–41]. The 20 Hz pulses occur at regular intervals, measured by the inter-pulse interval (IPI); the spacing of the pulses is regionally specific [16, 19–21, 24, 28, 29, 36, 37, 42]. The combination of overtone frequency, IPI value, and pattern make a song type which can be used to aid in differentiating among populations of fin whales, even those that occur in close proximity [16, 20, 37].

The highly productive waters in the Southern Ocean support large aggregations of krill, creating an important feeding ground for a variety of marine mammals and birds [43–45]. One such species is the fin whale. In recent years, large aggregations of fin whales have been observed in the Western Antarctic Peninsula (WAP) [46–50]. This area is suggested as an important feeding ground for Antarctic fin whales, with an abundance of krill to feed upon [43, 44, 46, 51].

Fin whales typically undertake annual migrations from low-latitude breeding grounds in winter to high-latitude feeding grounds in summer [52], although certain individuals and populations have been observed on feeding grounds year-round [53]. While complete migration patterns for WAP fin whales are not yet fully understood [54], recent studies suggest these fin whales migrate to the coast of Chile during the winter [51]. While they are generally present off WAP beginning in austral spring [44], their songs have previously been recorded from February through July with a peak in May [28, 51, 55–57]. This seasonal occurrence of song is likely another indicator of its function during reproduction, as fin whales are known to engage in mating in late autumn and early winter [51, 52]. Antarctic fin whales have been considered as a single circumpolar population [54], but analysis of song could prove a useful mechanism to differentiate between different breeding stocks [16, 19–21]. Antarctic fin whales in different parts of the Southern Ocean produce overtones occurring at different frequencies, which has resulted in the suggestion it could serve as a possible population delineator [28, 39]. However, to our knowledge no previous work has been done to describe the IPIs of Antarctic fin whale song. To this end, we have examined the seasonal and annual trends in these song characteristics for fin whales in the WAP from 2001–2004 and 2014–2016. The two recording periods were then compared to determine the stability of fin whale song over time in this area, thus evaluating its feasibility to serve as a population structure indicator.

## Materials and methods

**Fig 1:**
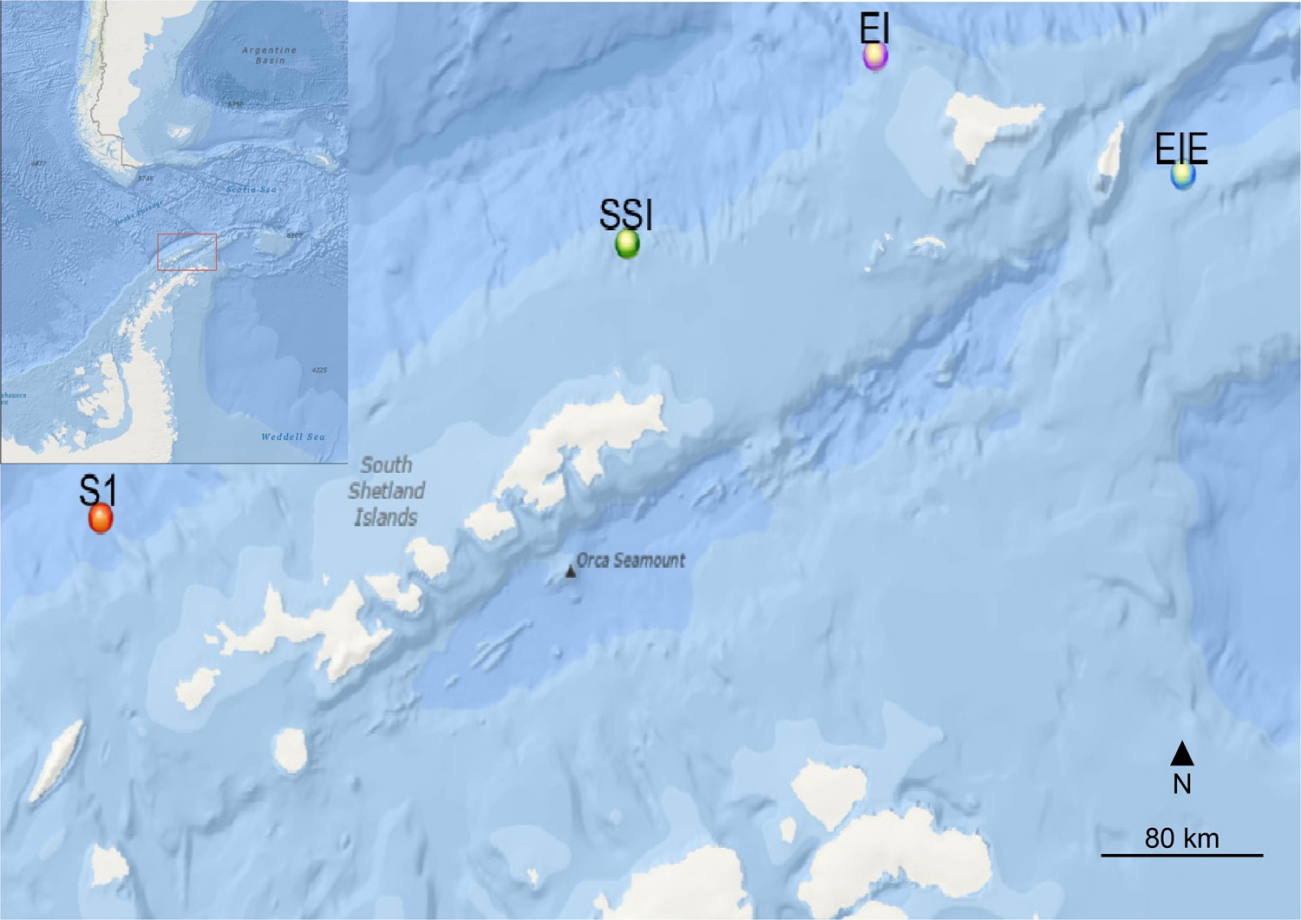
Study area map. Acoustic recorder deployment sites: Site 1 (S1, red) was used 2001–2003, Elephant Island (EI, purple) in 2003 & 2014, South Shetland Island (SSI, green) in 2015, and Elephant Island East (EIE, blue) in 2016. Inset map in top left corner shows study area in red square. Map images were obtained from USGS National Map Viewer.

Passive acoustic recorders were deployed off the coast of the WAP from 2001–2003 and 2014–2016. Previous studies have analyzed seasonal fin whale acoustic presence using recordings from 2001–2003 [55]. Earlier recordings were collected using Acoustic Recording Packages (ARPs) [58] while High-frequency Acoustic Recording Packages (HARPs) [59] were deployed from 2014 through 2016. Both versions of recorders were outfitted with data loggers and batteries which were moored to the seafloor with a hydrophone suspended approximately 10 m above. Deployment locations moved among four sites that were within 300 km of each other in the archipelagos at the north end of the WAP: Site 1 (S1) in 2001 through 2003, Elephant Island (EI) in 2003 and 2014, South Shetland Islands (SSI) in 2015, and Elephant Island East (EIE) in 2016 (Fig 1). No permits were required, and all fieldwork was performed in accordance with relevant federal and international regulations.

During all deployments the data were recorded with 16-bit A/D quantization. The ARPs had an effective bandwidth of 250 Hz. Deployments from 2014–2016 had higher sampling rates, resulting in an effective bandwidth of 100 kHz. These HARP recordings were decimated by a factor of 100 to create a dataset with an effective bandwidth of 1 kHz for easier review. In 2014 and 2015 recordings were duty-cycled with recording periods of 5 min out of every 6 and 10 out of 13.5 min, respectively, but data were recorded continuously in all other years (Table 1).

**Table 1:** Recorder deployment details.

Site	Lat (S)	Long (W)	Depth (m)	Record Start	Record End	Duty cycle
Site 1 (S1)	62˚16.44’	62˚10.02’	1600	March 23, 2001	February 9, 2002	N/A
				February 28, 2002	February 28, 2003	
				February 28, 2003	February 29, 2004	
Elephant Island (EI)	60˚53.21’	55˚57.24’	762	January 13, 2003	April 21, 2003	N/A
				March 5, 2014	July 14, 2014	5/6
South Shetland Island (SSI)	61˚27.47’	57˚56.52’	768	February 10, 2015	January 29, 2016	10/13.5
Elephant Island East (EIE)	61˚15.11’	53˚29.01’	1033	February 3, 2016	December 2, 2016	N/A

Location, depth, and recording durations for each deployment, including duty cycle (recording duration/cycle interval in minutes; N/A means recording was continuous).

Recordings were spectrographically visualized using the custom MATLAB (MathWorks, Natick, MA) software program *Triton* [49]. Long-term spectral averages (LTSAs) with 5 s time and 1 Hz frequency resolution were calculated from the recordings to facilitate review of data. To examine long-term trends in fin whale song characteristics, two days per month were randomly selected to be analyzed, one before and one after the 15^th^ of the month. This protocol allowed for a minimum of two whale observations per month, although if multiple song sequences were present in a given day it was not important for our analysis whether they were produced by a single or multiple animals. Data from the selected date were manually reviewed for presence of clearly identifiable fin whale songs. Identifiable song was defined as periods of fin whale 20 Hz pulses visible in 1 hour LTSA view that revealed a clear and consistent IPI pattern upon closer inspection via spectrogram. When evidence of song was found, these periods were examined using 120 s spectrograms with a frequency range of 0–150 Hz with 0.1 s time and 1 Hz frequency resolution. Periods of intense 20 Hz pulse production, when individual songs could not be identified, were excluded from analysis. For the purposes of this analysis, song was defined as periods of patterned 20 Hz pulses that persisted for two minutes or longer, in keeping with the protocol used for previous fin whale song analyses [16, 29]. A song was considered to end if no further pulses were observed within 60 s of the last observed pulse; the next observed pulse was considered the start of a new song. Each 20 Hz pulse in a song was logged, extracting measurements of start time and peak frequency of overtones.

After a day was fully reviewed, inter-pulse intervals (IPIs) were calculated as the difference in start time between one 20 Hz pulse and the next one. Each song bout was then categorized as either a singlet, doublet, or triplet. We defined a singlet as a song containing a single, consistent IPI value. A doublet contained two distinct, alternating IPI values whereas a triplet had two distinct IPI values that can be produced in a consistent pattern of long-long-short (long triplet) or long-short-short (short triplet) [16]. In the event no song was detected on the chosen date, surrounding days from the same half of the month were analyzed until song was detected or the month-half was fully reviewed and no evidence of song was found.

Each day analyzed was manually annotated to determine song type based on the distribution of IPI values. If a song contained a single-mode IPI it was considered a singlet. A song with a bimodal IPI distribution was considered a doublet or triplet based upon prevalent patterning of those IPI values. For each song variant present, the median and first and third quartile IPIs were calculated for each distinct IPI mode. IPI values were characterized by median instead of mean due to the non-normal distribution of IPI values. To determine whether IPI values changed over time, median IPI values were compared across years using a nonparametric Kruskal-Wallis test; years were compared post-hoc using the Dunn method [60] and adjusted by the Benjamini-Hochberg false-detection rate (FDR) method to correct for multiple comparisons [61]. Annual mean frequency of the overtones was calculated and effect of year on mean overtone frequency was determined using the same tests. Seasonality of each song variant was analyzed by comparing the proportion of each song variant by month. This proportional value was calculated by dividing the sum of pulses classified to a certain variant type in a particular month across all years by the sum of all pulses logged that month across all years. To examine how song variant preference changed over time we compared yearly song variant dominance. Song variant dominance each year was determined by comparing relative proportion of pulses for each variant, calculated as the number of pulses classified as a particular variant relative to the total number of pulses that year.

## Results

**Fig 2:**
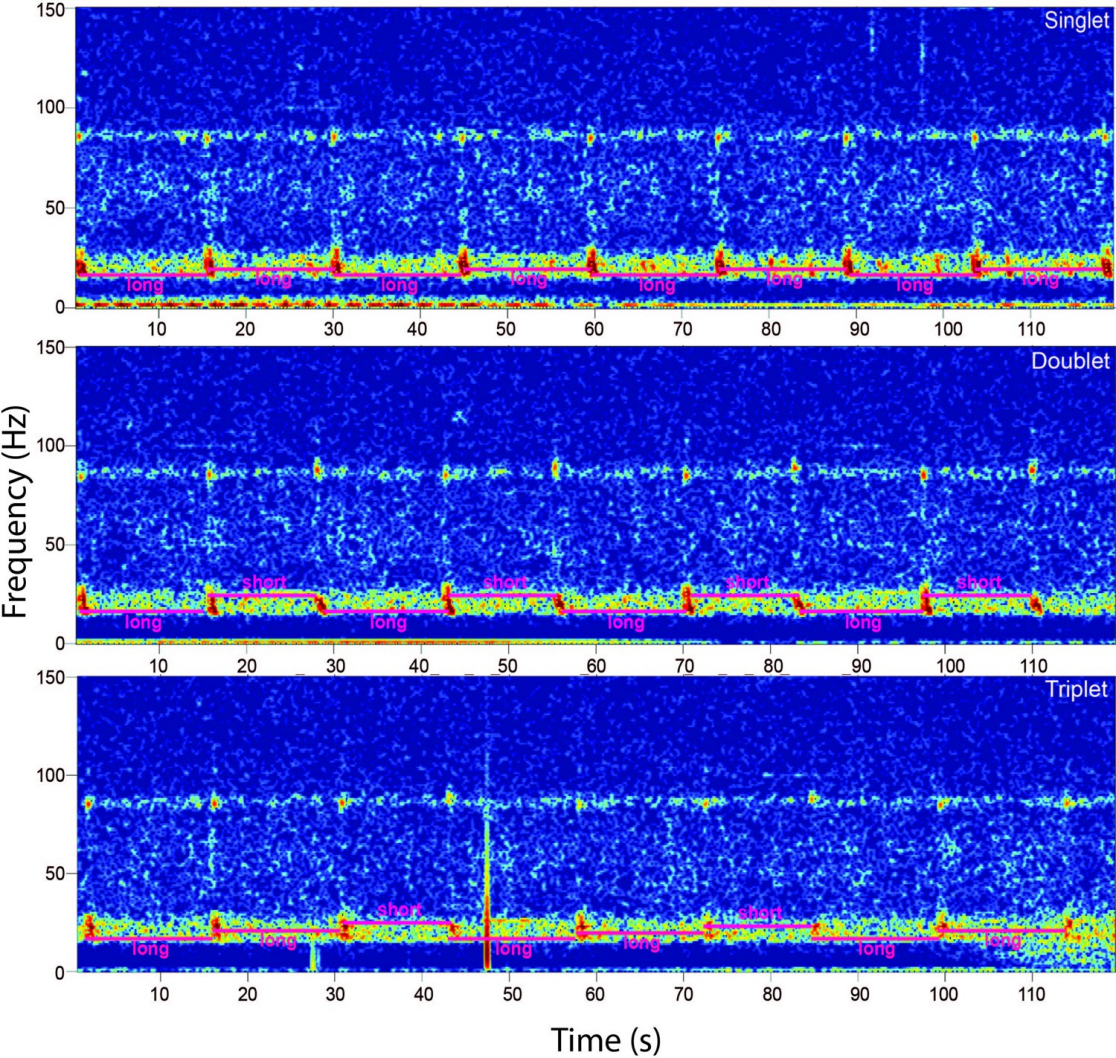
WAP fin whale song. Two-minute spectrograms recorded in the vicinity of South Shetland Island on April 2, 2015, depicting patterned fin whale 20 Hz pulses with accompanying overtones. The spectrograms display all three recorded song variants: singlet (top), doublet (middle), and triplet (bottom). The spectrograms were calculated using 2000-point FFT, 90% overlap and Hanning window.

**Fig 3:**
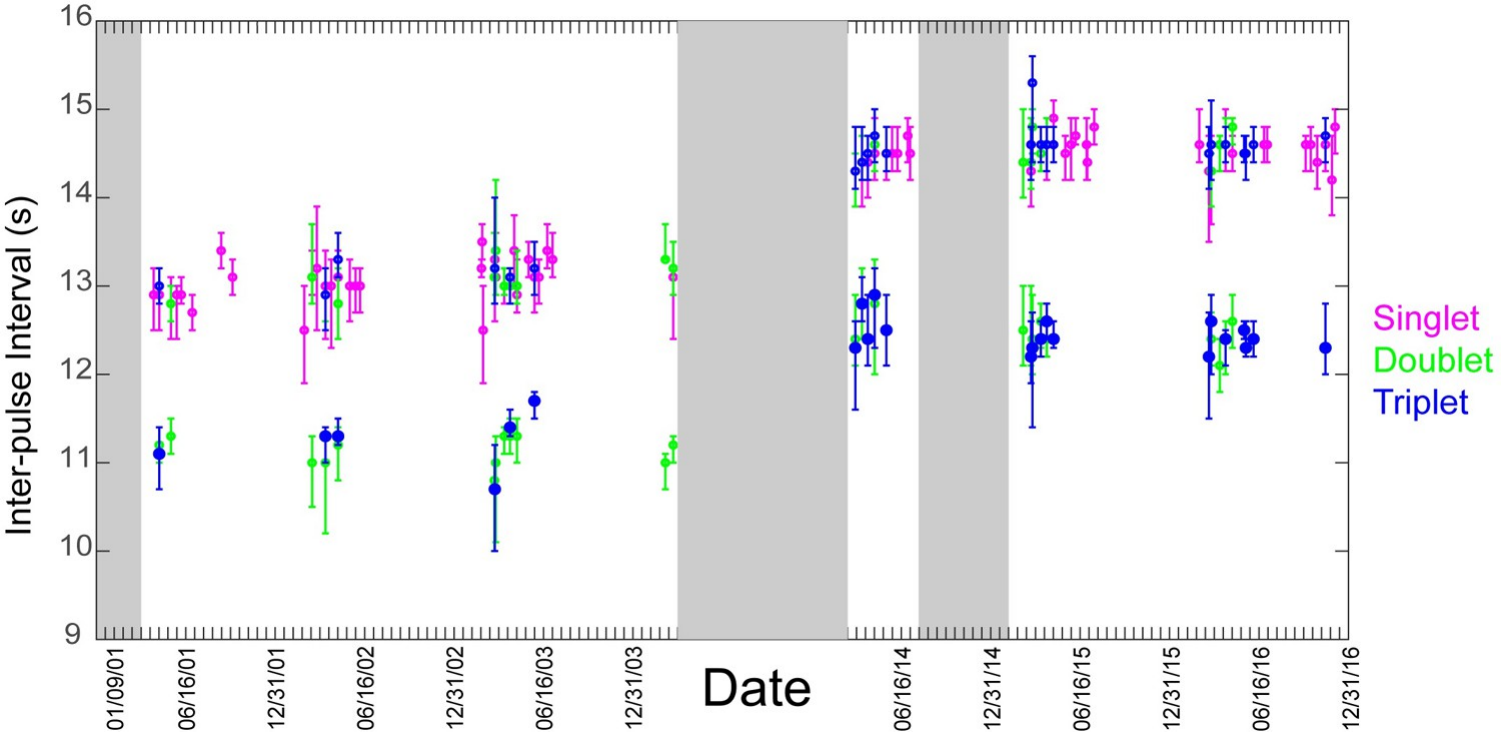
Daily median fin whale song inter-pulse interval (IPI). Plot of daily median song IPI, with error bars representing first & third quartiles. Each datapoint summarizes the song from a randomly selected date from each half-month. Singlets are in pink, doublet variants in green, and triplets in blue. Gray-shaded areas indicate breaks in recording effort. Dates on the x-axis are reported in mm/dd/yy format.

**Fig 4:**
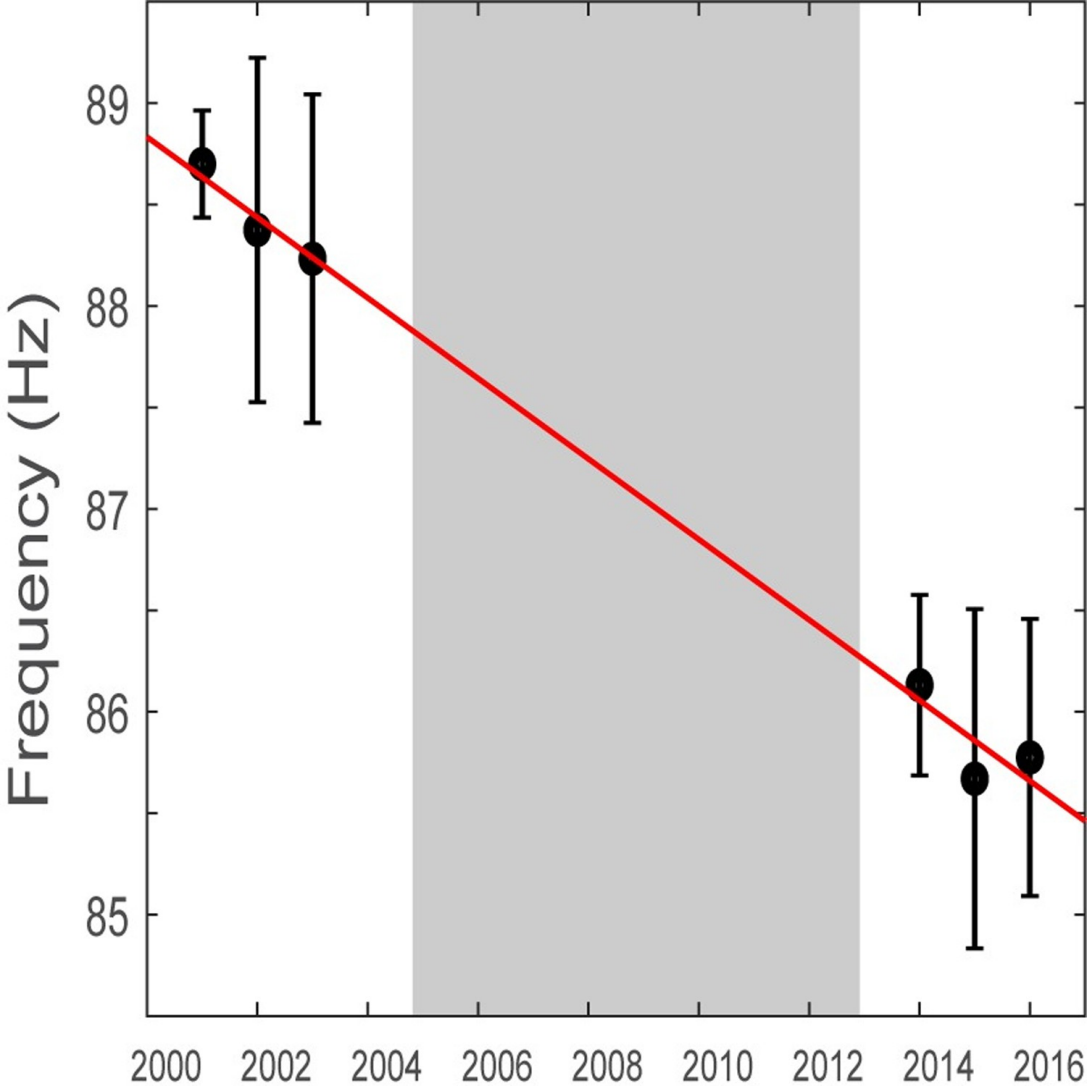
Annual mean overtone frequency. Mean overtone frequency (circles; in Hertz) and standard deviation (bars) of fin whale pulses for each year analyzed. Red line represents linear trend. Gray-shaded area indicates a break in recording effort.

Based on its IPI values and associated overtone frequency, there was one fin whale song type detected in the WAP with overtones present below 90Hz. The primary ‘long’ IPI value was approximately 14 s, two seconds longer than the secondary ‘short’ IPI which was about 12 s during the latter part of the study. Singlet, doublet, and long triplet variants were all observed (Fig 2). We considered the variants to be part of a single song due to the consistency of overtone frequency and IPI values amongst song types within the same year. The interval between pulses was significantly affected by year (K = 66.64, p<0.001). The primary IPI increased from 13 s in 2001–2004 to 14.5 s in 2014–2016 while the secondary IPI increased from 11 s to 12.5 s (Fig 3). The overtone frequency significantly decreased over the years (K = 57.29, p<0.001) from 89 Hz in 2001–2003 to 86 Hz in 2014–2016 (Fig 4).

**Fig 5:**
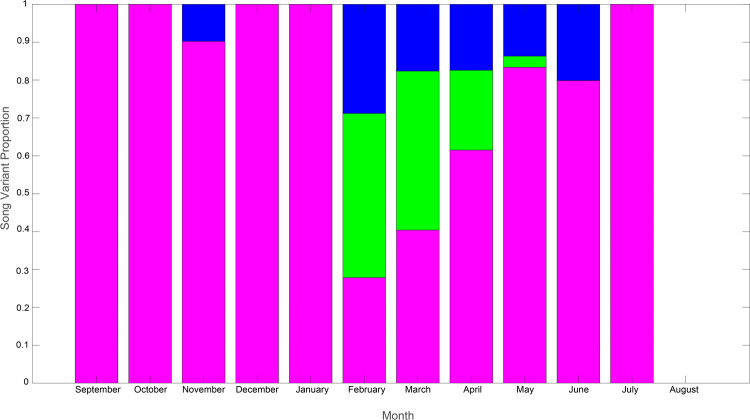
Monthly song variant proportions. Proportion of pulses by month over the course of a year (September to August) belonging to each call variant. Singlets are in pink, doublets in green, and triplets in blue in each stacked bar.

Song was recorded in all months except August, however it was only present in all recordings from April through June (S1). Recordings from 2016 contained the only observations of song from October through December and song was only present in January on January 31, 2003. The lack of song in February 2002 and March 2001 could be due to shorter recording effort during this time. Song was present in July in 45% of recordings and in September during 30% of recordings. The singlet variant was generally the most common IPI variant throughout the year and song was most complex February through May (Fig 5). Doublets tended to occur in the late austral summer and early autumn, February through April, while the triplet variant persisted into the early winter, February through June.

**Fig 6:**
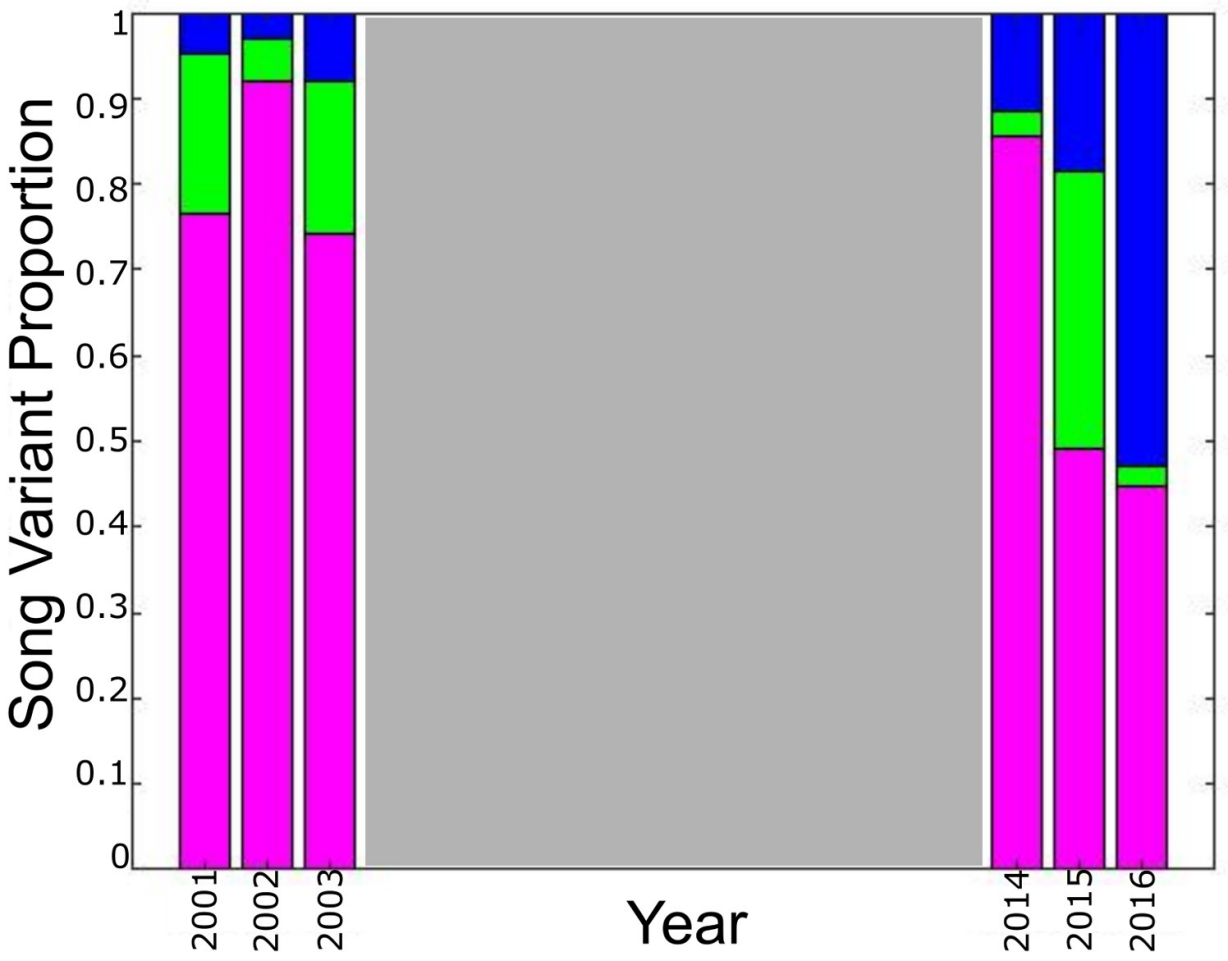
Annual song variant proportions. Proportion of pulses per annual recording effort belonging to each call variant. Singlets are in pink, doublets in green, and triplets in blue.

The singlet variant was prevalent during 2001–2003 and 2014–2015 (Fig 6). Data from 2004 were excluded from this analysis as recording only occurred in January and February. In early recordings triplets were least common, accounting for less than 10% of songs in 2001–2004. By 2016, however, the most prevalent variant type was the triplet song, which accounted for 53% of all song occurrence.

## Discussion

### Song description

Previous research of fin whale 20 Hz pulses in WAP characterized the song as a series of pulses with a duration of less than 1 s that sweep from 28 Hz down to 15 Hz [55] and are often accompanied by overtones with a frequency under 90 Hz [28]. However, this study is the first to describe the IPI patterning. A single song type was observed off the WAP coast, based on IPI values and overtone frequency. Within that song three variants were observed. The singlet variant contained only the primary long IPI value while doublet and triplet variants patterned the primary long IPI with a secondary short IPI, which was shorter by 2 s. While both short triplets and long triplets are possible only long triplet patterns were observed in the data.

The IPI of the song lengthened by 0.1 s annually while overtone frequency decreased by 0.2 Hz per year. Similar changes in fin whale songs have been reported in other ocean basins [16, 31, 35–37]. It should be noted that while these small changes are at the edge of the time resolution and smaller than the frequency resolution sensitivity of spectrogram measurements in this study, trends in the data were detectable due to the long-term nature of the data. Studies using shorter time scales should consider using finer-scale measurement parameters to detect changes of this scale. While the cause of these shifts are not yet clear, several theories have been suggested.

Whales could be compensating for increasing anthropogenic noise. Baleen whales have been observed to alter calling behavior in response to changing noise conditions [62, 63], however this is not an entirely satisfying explanation since song shifts have been observed across ocean basins despite wide variability in anthropogenic inputs [57]. Shifts in song characteristics could also be due to increased mating competition as populations recover in a post-whaling era [33]. Changes in call rate have been observed in songbirds [64] and frogs [65] in response to competitor presence, this behavior could be present in whales as well. While this hypothesis could be pertinent for Antarctic fin whales, since there is some evidence of increasing population [44, 51], not all fin whale populations and other whale species exhibiting frequency shift are experiencing the same population growth. Changes to song might also be the result of cultural drift, as baleen whale species have demonstrated vocal learning capability [9, 12, 66]. This has been extensively studied in humpback whales, which can change their song slowly over the course of a singing season [10, 15, 18, 23] and also abruptly when introduced to foreign singers [17]. Cultural drift can be caused through either the accumulation of errors or through innovation [3]. Computer simulations suggest that vocal learning to create population-specific songs requires low error rates in learning and low individual mortality [67]. The observed temporal and frequency changes have small enough annual rate of change that they likely lie outside of the perceptible range for these whales [68], so changes are more likely to be due to error than innovation. However, the observation of similar changes across ocean basins and species makes this explanation unlikely. To help explain the mechanism by which isolated populations change aspects of their song in similar ways, other researchers have suggested that whales follow a universal set of rules dictating how song changes over time [18]. How these universal rules might be encoded is unclear; genetic, cultural, and physiological mechanisms are all possible.

In addition to shifts in overtone frequency and IPI length, changes in song complexity were also observed. While variants were present each year of recording, there was a shift in variant occurrence. Singlet song tended to be prevalent, however in later years the proportion of triplet song variants increased, with the highest proportion occurring in 2016. Since song is presumed to serve a reproductive function [2, 8, 11], it is possible that this shift was motivated by increased competition due to an increased population size following the cessation of whaling [8, 44]. In several species of birds, females have demonstrated a preference for males who sing more complex song [69–72]. A similar trend has been observed in mice [73]. The ability to sing more complex song is theorized to indicate better fitness [69–71]. Alternatively, if different variants convey specific environmental information [16, 42], it is conceivable that environmental conditions were shifting in later years and by 2016 were favorable for increased use of the triplet song variant. Analysis of future recordings to determine variant preference after 2016, in conjunction with measures of prey and oceanographic conditions, would be needed to determine whether this shift in song variant prevalence may be related to environmental factors or if it is an indication of more long-term sexual selection pressures.

### Seasonal trends in song complexity

The song in this area was relatively stable within each year, with no observable change in IPI values over the course of one singing season. This contrasts with the observations of other studies, which have found some populations of fin whales change IPIs during a single year [29, 35, 37, 42]. In WAP, we did not observe this change in IPI, instead it was the complexity of the song that appears to change over the course of a singing season.

When fin whales arrive in Antarctica in austral spring, September through November, song is almost entirely comprised of the singlet variant. This remains true in early summer, December and January. This tendency to favor singlets abruptly changes in February, when all three song variants appear and no one variant constitutes more than 50% of pulses. The presence of all three song variants persists through May, although singlet song returns to dominance in April. Triplet song persists in June, but by July only singlet song remains. No evidence of song was found in August. It is interesting to note that song is most complex, i.e. contains the greatest level of variants, in February while previous studies have found peaks in acoustic presence in May [28, 51, 55–57].

The offset between peak song complexity and peak song production suggests that the complexity of song in this time cannot be explained solely by an increased number of singers. Given this fact, and the differing seasonality trends of the three variants, it is unlikely that the three variants are the result of different individuals that sing one variant type exclusively. It appears more probable that individuals switch between variants depending on context. The mechanisms driving variant use are not yet known. Perhaps these different variants serve unique functions within the population; others have posited that intra-annual changes in song might serve the purpose of conveying changes in prey abundance or other environmental factors [16, 42]. Seasonal changes to song alternatively might also be driven by changes in relative competition for mates or triggered by changes in hormone production [29].

### Using song as a population indicator

The song of WAP fin whales remained relatively stable over the course of this study. The decrease in song frequency by 3 Hz over 15 years, which averages a change of -0.2 Hz/year, agrees with previous reports of declining fin whale song frequency from the North Pacific and measured by the frequency of the 20 Hz pulse [31, 35]. Other studies have also reported lengthening IPI in song over time, from +0.58 s/year [35] to +1 s/year [36] while the IPI of song in WAP appears to change at a rate of +0.13 s/year. These changes tend to occur in small enough increments that the song is recognizable from year to year. A study in southern California and the Gulf of California documented abrupt fin whale song changes [16] whereas as recordings from Washington documented a gradual shift from one song type to another [35]. Abrupt changes in song type have been suggested to indicate that a new population has moved into an area [16] while gradual shifts could be the result of innovation or the introduction of foreign individuals whose songs are slowly adopted by the greater population [35]. The lack of such changes in the data presented here may provide preliminary evidence of a single, stable population of fin whales in the Western Antarctic Peninsula that has been consistently using the area that is rarely exposed to foreign individuals.

The frequency of overtones has been used to separate different populations of Antarctic fin whales [28, 39]. The Western Antarctic population has an overtone first described at 89 Hz [28], although our findings demonstrate a downward shift to 86 Hz by 2016. In Eastern Antarctica, a population of fin whales singing with a 99 Hz overtone has been reported west of 115°E [28, 39, 74]. There is also evidence of a second Eastern Antarctic fin whale population that had two distinct overtones at 82 and 94 Hz [39]. All recordings from Antarctica report similar bandwidth for 20 Hz pulses, 30 to 15 Hz, and these pulses tend to peak in austral autumn [28, 51, 55–57]. This agrees with our finding of consistent fin whale song from April through June, and with current understanding of fin whale migration patterns [52]. The migratory pathways for these different populations are not yet fully understood, however some hypotheses have been offered.

A migratory link between the WAP and Chilean waters has been proposed previously based on the presence of overtones at approximately 85 Hz, which matches closely the overtone observed in more recent recordings in this study [51, 75, 76]. A dominant IPI of 14.4 s from 2007 to 2016 was also reported off Chile [75], which agrees well with the song described here for the Western Antarctic Peninsula. The song in this area tends to peak in austral winter [75, 76], when previous studies note a decline in song occurrence in Antarctica [28, 51, 55–57]. In Eastern Antarctica, two separate populations have been proposed based on distinct overtone frequency values [39]. This hypothesis is supported by recent studies of fin whale acoustic presence off Australia’s east and west coasts [77]. Fin whales arrive off the coast of Western Australia in April and off Eastern Australia in June, therefore it has been hypothesized the two coasts represent separate populations. The arrival of fin whales to Western Australia in April, with peak song occurring in August, agrees with studies off Antarctica that found evidence of song in more westerly Eastern Antarctica from March through June [28]. To our knowledge no analysis of seasonality trends for the more easterly East Antarctica song exists to compare to the Eastern Australia patterns, where fin whales tend to sing most in May. However, a doublet song with two distinct overtones, around 80 and 90 Hz, was reported in Cook Strait off New Zealand in February through September of 2017, with peak singing occurring in May [40]. A different doublet song with overtones at approximately 70 and 80 Hz was recorded off the coast of Gisborne, New Zealand in 2014–2015 with peak detections in July.

To better understand the migratory patterns and population structure of Antarctic fin whales, recording effort and analysis should be increased in the southern hemisphere. Further recording effort and acoustic analysis should be performed in Eastern Antarctica and Australia and New Zealand to characterize song and determine whether migratory linkages can be made. Additionally, the deployment of acoustic tags throughout the Southern Ocean on male fin whales, equipped with satellite tracking, would enable researchers to track movement and calling behavior of individual whales [78]. In some instances, it has been possible to confirm genetic differences between populations with unique songs [19]. Similar genetic studies in the southern hemisphere would further increase our confidence in the utility of song as a tool to distinguish between distinct populations. The ability to delineate between genetically distinct groups would be particularly useful in Antarctica, where fin whales are currently managed as a single circumpolar stock [54] and environmental conditions necessitate remote monitoring [50, 78].

The use of passive acoustic monitoring methods is especially attractive in environments as challenging as Antarctica [49, 50]. The ability to detect animal presence over long time scales, even in remote areas often obstructed by sea ice, allows researchers to more fully understand annual presence cycles and how acoustic behavior can change over time. There are, however, some limitations which must be accounted for in interpreting this type of data. Chiefly, this approach only detects animals that are producing sounds. Further, some calls are produced by only a certain segment of the population or during specific times of the year. Fin whale 20Hz pulses are produced only by males [26] and typically occur in summer and fall [28, 35, 51, 55]. As a result, it is often challenging to translate call counts into population estimates [79]. There is currently poor understanding of typical call rates for individuals and if the interval between individual song utterances is constantly changing, it might not be possible to estimate population densities simply from call count data. Thus studies such as this, which is the first attempt to our knowledge to examine how song preference changes seasonally in WAP fin whales, provide information that is helpful to further attempts to use song for population size estimation.

## Conclusions

The data presented here represent the first known detailed description of the WAP fin whale song. The 20 Hz pulses of this song are accompanied by overtones below 90 Hz. The primary long IPI of the song is 2 s longer than the secondary short IPI. While the song had a primary IPI of approximately 14 s and secondary IPI of 12.5 s, the IPI lengthened by 1.5 s and overtone frequency decreased by 3 Hz between 2001–2003 and 2014–2016 recording periods. Three variants of the song were recorded, with singlet song being most common. Song was most complex, i.e. contained the most variants, in early austral autumn. The song remained relatively stable over this 15-year period, which makes fin whale song an attractive tool to indicate population identity. Further research is needed to examine long-term trends in song variant preference and to observe whether IPI and overtone frequency drift persist. In addition, extending recording effort to other potential fin whale aggregations sites such as Eastern Antarctica, Chile, Australia, and New Zealand would allow for better understanding of migratory pathways in the southern hemisphere. The use of acoustic tags would allow for individual tracking and behavioral and environmental data may provide valuable clues to potential drivers of song preference. Genetic analysis of fin whales, in combination with acoustic recordings from the southern hemisphere, would allow us to test whether fin whales singing the same song can be considered a distinct population.

## Supporting information

S1 Data Raw call data. (XLSX)
